# The Technique of Nerve Resections for the Relief of Pain about the Face and Jaws

**Published:** 1894-11

**Authors:** M. H. Cryer

**Affiliations:** Philadelphia


### THE TECHNIQUE OF NERVE RESECTIONS FOR THE RELIEF OF PAIN ABOUT THE FACE AND JAWS.[1]

BY M. H. CRYER, M.D., D.D.S., PHILADELPHIA.

Mr. President and Gentlemen of the Academy of Stomatology,—It gives me great pleasure to have the honor of presenting the first essay to this body for its consideration and discussion: the more so as the name of the society marks the recognition of a distinct evolutional advance in our professional relations. The intelligent practice of what has come to be considered within the legitimate province of the dentist to-day includes so much more than the mere technique of dental operations that a more comprehensive designation of the field is not only desirable but necessary.

---

[1] Read before the Academy of Stomatology, Philadelphia, October 6, 1894.

My gratification over the name "stomatology" for this society is, I believe, shared by every one of you. Indeed, the term might have been chosen some time ago, as the dentist has long since outgrown the limitations of his original field in which he devoted himself exclusively to the care and the treatment of the teeth. In the pursuit of his calling he naturally became more familiar with the functions of the mouth and its associate parts, until now he is fully equipped to deal with all complications connected with, or dependent upon, diseased conditions within his especial territory. And not only in his immediate department has this impress been made; many allied branches claim his devotion and intelligence. His trained mechanical ability has enabled him to produce the best microscopical sections of the different tissue connected with the mouth in all stages of their development. We are also proud to know that from our ranks have come the finest bacteriological studies of these regions that have been made. So brilliant has been the development of bacterio-pathology of the mouth as compared with general bacterial pathology that it presents the spectacle of a branch growing out of the parent stem. We are shortly to celebrate the fiftieth anniversary of the application of anæsthetics. This greatest of blessings to suffering humanity is due to the intelligence of Horace Wells, a dentist. In the mechanical department, the characteristic inventive spirit has been strongly manifested; instruments and methods are being constantly improved and new ones devised. The activity which has characterized the development of our knowledge of oral science, as just noted, is seen in all its departments. Our knowledge of pathological conditions and their rational therapeutics has made rapid strides until the great majority of morbid processes are fairly controllable by appropriate treatment. This general advance is, as a natural consequence, reflected in our educational methods, so that the equipment of the dentist of to-day is necessarily more thorough and satisfactory than was formerly possible. In view of these facts, it would seem to be quite within consistent limits to adopt the more comprehensive title by which the association is designated; a term which distinctively describes the broader sphere of activities, which is by common consent the legitimate field occupied by the dental practitioner of the present. The department of oral surgery is one which in my judgment has occupied a somewhat anomalous position in the past. The operative measures for relief of pain about the face and jaws have been conducted almost exclusively by general surgeons. It will hardly be gainsaid—certainly not by this audience—that but few general surgeons have the training necessary for producing the special manipulative skill demanded for this work. I mean by this the kind of manual dexterity which is the acquirement of the properly-trained dentist. I should be lacking in historical accuracy as well as in loyalty and due appreciation were I to omit paying just tribute to the achievements in oral and general surgery of one who, beginning his training in the ranks of dentistry, has by his natural abilities and special qualifications of heart and mind won fame for himself and honor to his calling. It is the class of operations so closely identified with the life-work of Professor James E. Garretson that will form the basis of my communication this evening. The subject proper being the technique of these operations, we may dismiss all consideration of reasons which compel them.

It is assumed that a diagnosis has been made of a lesion affecting a nerve-trunk; that we have excluded the possibility of the disorder having peripheral cause. We assume the existence of a lesion not peripheral, and will consider operations upon the great branches or trunks of the superior and inferior maxillary nerves. There is one problem which confronts the surgeon here, and that is the leaving of disfiguring cicatrices, a question which scarcely concerns those who operate upon parts of the body which are hidden. This necessitates or makes advisable that we operate with as small an external opening as possible, after selecting such place as will leave the least noticeable scar. Much of his work is done, therefore, in the dark, his sole dependence being the sense of touch combined with accurate or absolute anatomical knowledge. It will be seen from this that these operations require the most delicate skill combined with a refined technique. The instrumental means prior to the past ten years, as they are now with some, are found in tenotomy knives, mallets, chisels, directors, scissors, and saws. A reference to Carnochan's work will give much of the history of these operations. Descriptions may be found in Gross's, Agnew's, or better, in Garretson's surgery. You are presumably familiar with Langenbeck's operation upon the infra-orbital nerve; this consists in cutting the trunk just beyond the spheno-maxillary fissure by a tenotomy knife thrust in from the outer and lower margin of the orbit; following this by an incision below the line of the orbit to the infra-orbital foramen, where the severed nerve is caught and withdrawn. All other operations that I will describe, save this one, require bone section; for this there is no device approaching in completeness or effectiveness the improved surgical engine, with its perfected instruments, most of them evolutions of the dental-engine burs.

There are several operations for the resection of branches of these nerves which are of superficial relation exclusively; as they are comparatively simple, we will proceed at once to consideration of the deep sections. There are two principal operations for the resection of the superior maxillary nerve: one of these is in the posterior portion of the orbit, the other in front of and as near the round foramen as possible, this last involving the removal of Meckel's ganglion. The first operation is nicely accomplished by the following method, somewhat after Stoker: the eyelids should be closed by a single suture, then a curved incision made along the infra-orbital ridge down to the bone, then a vertical cut downward to the infra-orbital foramen. Spreading the soft tissue, remove the periosteum from the bone covering the canal, and secure the nerve with a ligature, then pass a dental nerve-canal plugger into the foramen over the nerve, as a director and guard, along the canal, until the point is in the orbit; by using a small surgical bur the bone of the roof of the canal can be quickly cut down to the protecting plugger, when the nerve may be lifted from the canal intact. Now pass the nerve with the attached ligature through the opening of a neurotome, which must be worked backward as far as possible; then, holding the nerve tightly, revolve the cutting portion, and the nerve will be severed close to the spheno-maxillary ganglion.

The second operation may be performed in two ways: the first and, to my mind, better one is to make a trap-like opening exposing the greater portion of the anterior face of the superior maxillary bone, find the foramen, and secure the nerve as before, then with the engine remove a portion of the face of the bone immediately below the foramen, thus opening the maxillary sinus. Control all hemorrhage as far as possible, and, again using the engine, open through the inner and superior portion of the posterior wall of the antrum, and enter the spheno-maxillary fossa, thus exposing the Meckel ganglion. By passing a long, delicate nerve-canal plugger below the nerve as a means of protection from the engine-bur, the bone of the floor of the canal may be cut away, using a long-shanked bur. Those unaccustomed to the use of a surgical engine could use in its stead a long slender chisel with one edge prolonged. This prolonged portion of the chisel will pass along the floor of the canal and act as a guide, while the chisel proper will cut away the floor. After the floor of the canal has been removed, the plugger may be withdrawn and the nerve dropped into the antrum and passed through the fenestrated opening of a neurotome large enough to include Meckel's ganglion; pass the neurotome backward to the round foramen, keeping the nerve tightly drawn, revolve the knife, and divide the nerve, then pull it away.

There are many operations for the resection of the branches of the inferior maxillary division; but I shall take only those requiring bone-cutting. Professor Garretson's plan for the resection of the nerve within the inferior dental canal is as follows: An incision is made in the soft tissue of the chin under the jaw so that the resulting scar will be hidden by the tissues under the border of the inferior maxilla. The tissues are now elevated so as to lift the cut upward in a line passing over the inferior dental canal, holding the lips of the wound apart with retractors; the bony covering of the canal is then exposed and ready to be removed. Originally Hay's saw, a mallet, and chisel were used in this operation. An improvement in this was using the ordinary dental engine and cutting two parallel lines with a small circular saw, and uniting the two lines at each end by boring the bone with a bur or small trephine, thus completing the section. The strip of bone may then be lifted from its position, thus exposing the nerve. As the more powerful engine and specially adapted burs were introduced, this operation of uncovering the canal could be better and more quickly accomplished. After the bone is removed the nerve may be lifted from the canal and a section taken from it, after which the soft parts are closed by interrupted sutures. The wound soon heals, leaving a small scar in such a position that it does not show, except upon close observation.

Professor Agnew's operation is to make a trap-like opening over the lower half of the ramus, remove a portion of bone by the use of a trephine, thus uncovering the canal, when a portion of the nerve may be removed. These operations frequently give relief for a brief period, but usually there is a recurrence of the pain. After resection nature endeavors to replace the lost tissues by similar new growths, which take their place, and frequently the bony tissue of replacement encroaches within the canal to a greater or less extent. These new nerve-fibres are liable to become entangled in the new bone formation; the pinching or pressure resulting from this may cause a recurrence of the neuralgia. At the present time it is regarded as more advisable to make the resection between the inferior dental canal and the base of the skull. If the canal and nerve could be obliterated so that the latter could not again grow into the bone, I believe a permanent cure could be made in some cases. This might be prevented by sealing the inlet to the canal by a gold plug, but as this might act as a foreign body, it is possible that a sterilized sponge packing might induce a secondary deposit which would obliterate the canal.

The third operation is another one of Professor's Garretson's, and is similar to that described by Professor Agnew, except it is made a little higher and opposite the inferior dental foramen, thus exposing the nerve at the opening of the canal, where it is seized by a forceps and then dissected out from the tissue, and severed as near as possible to the oval foramen. The difficulty in this operation is the lack of space; there is also much danger from hemorrhage, so that the operation is not as satisfactory as we could desire. Recognizing this, Professor Garretson adopted in its stead what is known as the Pancoast operation. This consists in making an opening through the soft tissue on a line with the lower edge of the zygomatic arch, separating the tissues until easy access is obtained to the coronoid process, and sawing it off. In this way both the inferior and superior maxillary nerves are accessible, and may be excised near their respective foramina of exit from the brain. The objection to this operation is that it causes too great a loss of the temporal muscle on that side. It is also difficult by such operation to gain access to the nerve as it enters the inferior dental canal, on account of the awkward position. The intrusion of adipose tissue into the incision and the difficulty in taking the inferior dental nerve up from this point suggested to me some four years ago that, instead of cutting away the coronoid process, the sigmoid notch might be deepened to the inferior dental canal. To do this, expose the bone, take a barrel-shaped surgical bur having a smooth or safe end, remove the bone almost to the foramen, after which lift out the nerve, artery, and vein with a tenaculum, then separate and secure the nerve by hæmostatic forceps above the point at which the mylo-hyoid nerve is given off; draw as much of the nerve from the canal as will come, and cut the mylo-hyoid nerve as near to its point of origin as possible. If the dissected portion of the nerve is long enough, pass it into the neurotome; if not, attach a ligature and pass it in in that manner, holding the nerve tightly, with the point of the neurotome in the direction of the oval foramen; when this instrument can be passed no farther, revolve the knife and cut it as in the other operations. I believe the Garretson operation last spoken of is the best plan, to remove the nerve at the oval foramen if the neurotome spoken of and hereafter described is used.

The proper amputation of the nerve at the oval foramen is one which involves no little difficulty at times by reason of the hemorrhage and the limitations imposed by the surrounding tissue, which interfere with direct vision. The bony structures offer an unyielding resistance to the use of any of the special instruments which have heretofore been used for the purpose. To obviate the difficulties which I have noted, and to furnish a means for the amputation of the nerve with certainty and precision at the exact point desired, I have devised an instrument which I call a neurotome. It is constructed of steel, and consists of an outer tube with a fenestrated end, the free extremity of which is rounded or tapered, and having on its inside surface, near the end, a projecting shoulder which is continuous around the inner periphery of the outer or canular tube. The inner cutting portion of the instrument consists of a steel spindle, which fits closely and smoothly within the outer tube, and has its free end fashioned into a tubular knife similar to a cork-borer or leather-punch. In use, after the nerve has been dissected out from its canal, its end is passed through the distal opening of the neurotome and out through the fenestrum in its side. The instrument is gradually pushed along the nerve, and worked in the direction of its foramen of exit. When it has been ascertained that the end of the neurotome has been brought into close contact with the bone at the foramen, and the nerve is made tense by traction, the central cutting-punch of the neurotome is driven home with a rotary motion against the shoulder mentioned before at the end of the outer canular tube, which will cleanly sever the nerve at the point desired, with no risk of injuring the deeper structures or the adjacent blood-vessels.

Since first describing this neurotome, Dr. Thomas G. Morton, of this city, has shown me one of his devising; the knife, however, is on an outer working tube instead of inside. The cutting-edge abuts upon a projecting shoulder from the fenestrated end of the inner tube.

The operation of deepening the sigmoid notch was reported by me in August, 1892, at the American Dental Association. It was published in the November number of the *Dental Cosmos* of the same year. The operation had been performed twice at the Medico-Chirurgical Hospital before I reported it. In the English *Medical Times and Gazette* for December, 1892, Mr. Victor Horsley, M.R.C.S., reported his operation of deepening the sigmoid notch, the principle being the same, the cutting of the bone being accomplished by different mechanical means. After an interview with this gentleman, a few weeks since, when he made a minute examination of the improved surgical engine, he expressed himself as thoroughly satisfied as to its utility in simplifying bone operations. Mr. Christopher Heath, M.R.C.S., also expressed himself as much pleased with the Yankee invention. It has been my desire in bringing to your notice this cursory study of the technique of nerve resections, to especially call your attention to the marked improvements in method which are the direct outgrowth of the application of dental thought, ingenuity, and inventiveness. If by so doing I have in some degree led you to share my belief that oral surgery is a department, which, regarding the best interests of humanity, should be solely intrusted to the care of the properly-trained stomatologist, I shall feel more than gratified.

